# Retinoic Acid Neutralizes the Effects of Herpes Simplex Virus Type 1-Infected Cell Protein 0 (ICP0) in Retinal Pigment Epithelial Cells

**DOI:** 10.7759/cureus.61089

**Published:** 2024-05-26

**Authors:** Merve Sen, Özgür Eroğul

**Affiliations:** 1 Department of Medical Services and Techniques, Suhut Vocational School of Health Services, Afyonkarahısar Health Sciences University, Afyonkarahisar, TUR; 2 Department of Opthalmology, Faculty of Medicine, Afyonkarahisar Health Science University, Afyonkarahisar, TUR

**Keywords:** keratitis, retinoic acid, icp0, tlr3, hsv-1

## Abstract

Background: Herpes simplex virus (HSV) infection of the cornea, uvea, and retina is the leading infectious cause of blindness worldwide. This study examined the effects of retinoic acid (RA) on the protein levels of interleukin (IL)-17A and IL-23 cytokines with known proinflammatory effects and toll-like receptor 3 (TLR3) messenger RNA (mRNA) expression in retinal pigment epithelial (ARPE-19) cells treated with HSV-1-infected cell protein 0 (ICP0).

Methodology: We used 3-[4.5-dimethylthiazol-2-yl]-2.5-diphenyl tetrazolium bromide assay to calculate the half maximal inhibitory concentration (IC50) doses of RA and ICP0 in ARPE-19 cells. At the end of 24 hours, protein levels of IL-17A and IL-23 were analyzed using enzyme-linked immunosorbent assay. TLR3 mRNA expression levels were also calculated using reverse transcription-polymerase chain reaction (RT-PCR).

Results: RA administration decreased IL-17A levels, which were elevated by ICP0. The IL-23 levels were similar between the ICP0-treated and control groups, but the difference was significant between the ICP0-treated group and RA+ICP0 combination. These results showed that RA can significantly increase IL-23 levels in the presence of ICP0. Although ICP0 dramatically increased TLR3 mRNA expression compared with that in the control group, the RA+ICP0 combination returned TLR3 mRNA expression to a level similar to that in the control group (*P* = 0.419).

Conclusions: RA may potentially neutralize HSV-1 ICP0 negative effects in ARPE-19 cells.

## Introduction

Herpes simplex virus type 1 (HSV-1) is a linear, double-stranded DNA virus with a genome size of 152 kb [1] and the leading cause of infectious blindness worldwide. Primary eye infections are often asymptomatic and self-limiting. However, if viral pathology develops, HSV-1 presents follicular conjunctivitis, superficial punctate keratitis, and dendritic ulcers, the most defining features of ocular infection [2]. Herpes simplex infection is highly prevalent, with an estimated 67% and 11.3% of the global population aged 0-49 years exposed to HSV-1 and HSV-2, respectively. In developed countries, the incidence of herpes simplex keratitis (HSK) is between 10 and 30 per 100,000 population annually, with a prevalence estimated at 149/100,000 population [3]. Antiviral drugs may suppress symptoms caused by reactivation but do not eliminate the latent virus. Furthermore, drug-resistant viruses may develop with the long-term use of antiviral medications, such as acyclovir (ACV), valacyclovir (VCV), and famciclovir (FCV) [4]. Therefore, alternative treatments are needed for HSV-1 infections in patients resistant to therapy with ACV and its derivatives.

Mossman and Smiley described ICP0 as an early HSV protein that inhibits the antiviral effects of interferons [5], possesses E3 ubiquitin ligase activity, and plays a significant role in latent HSV-1 infection [6]. In vitro studies have shown that HSV infection activates the interferon signaling pathway in various cell types, and infected cell protein 0 (ICP0) plays an important role in inducing type 1 interferon resistance [7].

Interleukin (IL)-17 plays both pathogenic and protective roles in various viral infections. It induces neutrophil migration to the infection site, provides survival signals to neurons, and mediates immunoglobulin M (IgM) production through NF-κB and Blimp-1-mediated response pathways. Conversely, dysregulated IL-17 levels can lead to viral pathologies. IL-17 production induces hyperinflammation and leads to the release of matrix metalloproteinases and oxyradicals, resulting in tissue damage and tissue integrity disruption [8]. Additionally, IL-23 plays a crucial role in various immune responses, including differentiation, survival, and expansion of Th17 cells, γδ T-cells, and neutrophils [9,10].

Retinoic acid (RA), an active metabolite of vitamin A, is a potent immunomodulator, supporting immunotolerance by inhibiting Th17 cell differentiation [11]. However, RA can enhance inflammation by supporting effector Th1 and Th17 cells in inflammatory conditions [12,13].

## Materials and methods

The effects of RA on the protein levels of IL-17A and IL-23 and toll-like receptor 3 (TLR3) mRNA expression in retinal pigment epithelial (ARPE-19) cells treated with HSV-1 ICP0 were determined experimentally. The study groups were defined as control, ICP0, RA, and ICP0+RA combination.

Cell culture

ARPE-19 cells (American Type Culture Collection; CRL-2302, Manassas, VA) were incubated in Dulbecco’s Modified Eagle’s Medium supplemented with 10% (v/v) heat-inactivated fetal bovine serum, 5 mM glutamine, 100 U/mL penicillin, and 100 µg/mL streptomycin at 37 °C in an atmosphere containing 5% CO_2_ and 95% air. To grow the cells, approximately 3 × 10^6^ cells in 20 mL of growth medium were seeded into 75 cm^2^ flasks at a cell density of approximately 4 × 10^4^ cells/cm^2^. The flasks were then incubated for two to three days.

IC50 dose determination was performed with a 2,5-diphenyl-2h-tetrazolium bromide (MTT) cell viability measurement test.

From a cell suspension prepared at a concentration of 2 × 10^4 ^cells/mL, 100 μL suspension was transferred to each well of 96-well cell culture plates. The plates were incubated at 37 °C for 24 hours for the cell monolayer to cover the wells (50%-60%).

Dilutions of 1,000, 500, 250, 125, 62.5, 31.25, 15.62, 7.8, 3.54, and 1.5 µM were prepared for the commercially obtained lyophilized HSV-1 ICP0 (MyBiosource; catalog no. MBS1089623). They were applied to the ARPE cell line for 24 hours.

Dilutions of 500, 250, 100, 50, 25, 10, 5, and 1 µM were prepared for RA (TRC Canada; catalog no. R250000) and applied to the ARPE cell line for 24 hours.

After the 24-hour incubation period, 1 μL of MTT dye (5 μg/mL) was added to each well, and the cells were further incubated at 37 °C for two hours. The MTT solution was removed, and 200 μL of DMSO was added to each well, followed by incubation at room temperature (20-22 °C) in the dark for five minutes. The color change was determined using an enzyme-linked immunosorbent assay (ELISA) plate reader at a wavelength of 570 nm. Calculations were performed in the GraphPad Prism 9 program.

Total protein measurement

The cells were washed twice with Phosphate-buffered saline (PBS) and then harvested using a lysis buffer containing a protease inhibitor cocktail (Roche Complete, IN). Lysates were centrifuged at 16,000×*g* for 15 minutes at 4 °C. The BCA method (TaKaRa, Shiga, Japan) was used to determine protein concentration.

Determination of IL-17A and IL-23 protein levels using ELISA

IL-17A (eBioscience/LOT: 140783022) and IL-23 (eBioscience/LOT: 141697007) concentrations were determined using commercially available ELISA kits. Concentrations were measured using a microplate reader at 450 nm. The protein concentrations of the samples (pg/mg) were calculated using graphs obtained from standard concentrations.

Real-time polymerase chain reaction (RT-PCR)

GeneMATRIX UNIVERSAL RNA Purification Kit (EURx Ltd., Gdansk, Poland) was used for total RNA isolation. cDNA synthesis from isolated RNA was performed using the OneScript Plus cDNA Synthesis Kit (ABM, Richmond, BC) following the kit protocol. The obtained cDNAs were used in real-time RT-PCR experiments. Table 1 shows the primer sequences used. We measured cDNA concentrations spectrophotometrically (Epoch, BioTek, Winooski, VT), and we used an RNA/DNA ratio of ≥1.7. The appropriate cDNA volume was transferred to PCR tubes (AxygenP). Calculations were performed using a total reaction volume of 20 μL for each sample. Reaction mixtures containing cDNA were added to the wells. The PCR tubes were loaded into the CFX Connect, Real-Time System (Bio-Rad, Hercules, CA) instrument. We used the temperatures and cycles given in Table 1.

**Table 1:** PCR primer sequences of GAPDH and TLR3 for mRNA analysis. RT-PCR, real-time polymerase chain reaction; GAPDH, glyceraldehyde 3-phosphate dehydrogenase; TLR3, toll-like receptor 3

Genes [14]	Primer sequences (5′ → 3′)	RT-PCR programs	Cycle
GAPDH	F-5′ GATTTGGTCGTATTGGGCGC 3′ R-5′AGTGATGGCATGGACTGTGG 3′	95 °C-30 s/59 °C-1 minute/72 °C-30 s	35
TLR3	F-5ʹTGCACGGGCTTTTCAATGTG3ʹ R-5ʹACGAAGAGGCTGGAATGGTG3ʹ	95 °C-30 s/57 °C-1 minute/72 °C-30 s	35

The delta cycle threshold (ΔCt) values were calculated by subtracting the Ct values of each gene from the housekeeping Ct value of each sample. To calculate the delta-delta cycle threshold (ΔΔCt) values, the ΔCt values of each sample were subtracted from the Ct value of the control group. The 2^−ΔΔCt^ value was determined by obtaining the ratio of the final value to the initial value (fold change) to determine the expression level of each gene. Moreover, 2^−ΔΔCt^ values of >1 and <1 were interpreted as increased and decreased expression, respectively. These calculations were performed using the REST 2009 program. The glyceraldehyde 3-phosphate dehydrogenase (GAPDH) gene was used as the housekeeping gene.

Data analysis

The experiments were conducted in three replicates, and the mean values were evaluated using a one-way analysis of variance in the SPSS statistical program. Data were analyzed by using SPSS statistical software version 20.0. The presence of statistically significant study groups was examined with the one-way analysis of variance (ANOVA) for parametric. The comparison of groups for parametric was used Tukey’s multiple comparisons test. *P *< 0.05 was considered the threshold of statistical significance level.

## Results

Determination of IC50 doses for ICP0 and RA

**Figure 1:**
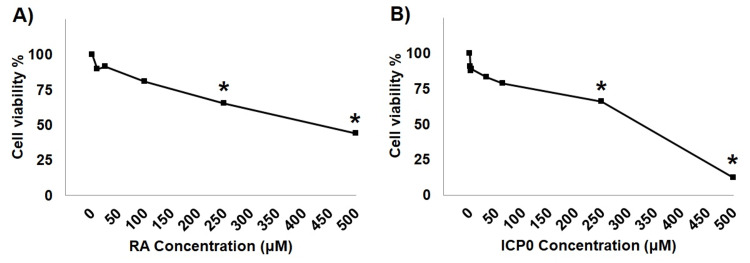
Determination of infected cell protein 0 (IC50) values for ICP0 and retinoic acid (RA). (A) IC50 dose concentrations of ICP0 and (B) IC50 dose concentrations of RA. The results are reported as means ± standard deviation of at least two independent experiments. (*) Differences were considered to be significant in experiments when *P* < 0.05.

The IC50 doses were determined as 424.3 and 277 µM for RA and HSV-1 ICP0, respectively (Figure 1). The cells were treated with the IC50 doses and incubated for 24 hours.

Determination of IL-17A and IL-23 protein levels using ELISA

The IL-17A and IL-23 protein levels were determined by administering ICP0, RA, and their combinations to the ARPE-19 cell line at predetermined IC50 dose concentrations and measured after 24 hours (Table 2).

**Table 2:** Alterations of the protein levels of IL-17A and IL-23 in ARPE-19 cell line after treatment with ICP0, RA, and ICP0+RA indicated. The cells were treated with 277 µM ICP0 alone (24 hours), 424.3 µM RA alone, and 424.3 µM RA plus and 277 µM ICP0 combination (24 hours), respectively. IL-17A and IL-23 groups were evaluated separately. Superscript letters indicate a statistical difference between the two groups (*P* < 0.05). ^a^Significance with regard to the control group. ^b^Significance with regard to the ICP0 group. ^c^Significance with regard to the RA group. C, control; ICP0, infected cell protein 0; RA, retinoic acid

Groups	C	ICP0	RA	RA+ICP0
IL-17A	289.4 ± 42.74	410.7 ± 41.65^a^	98.54 ± 5.748^a,b^	166.9 ± 26.01^b,c^
IL-23	13.131 ± 1816	9.816 ± 2040	21.972 ± 3487^a,b^	15.659 ± 238.7^b,c^

**Figure 2:**
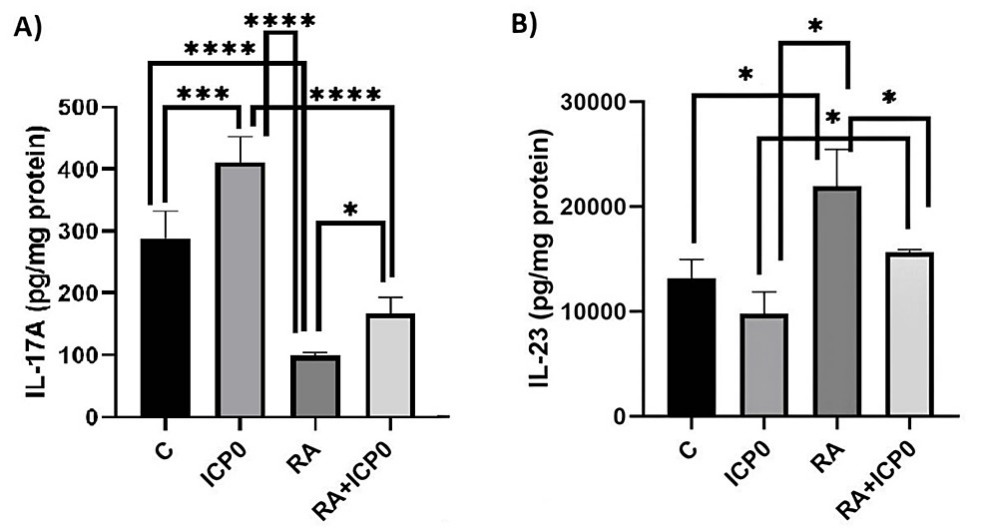
Alterations of IL-17A and IL-23 protein levels for different study groups. (A) IL-17A protein levels between the control (C), infected cell protein (ICP0), retinoic acid (RA), and ICP0+RA groups. (B) IL-23 protein levels between the C, ICP0, RA, and ICP0+RA groups. The results are reported as means ± standard deviation of at least two independent experiments. ^*^Differences were considered to be significant in experiments when *P* < 0.05. More stars indicate a smaller and more significant *P*-value.

The IL-17A levels in the control, ICP0, RA, and RA+ICP0 groups were 289.4, 410.7, 98.54, and 166.9 pg/mg protein, respectively. Additionally, the IL-23 levels in the control, ICP0, and RA groups were 13.131, 9.816, and 21.972 pg/mg protein, respectively (Figure 2).

IL-17A levels were significantly higher in the ICP0 group than in the control group (*P* = 0.001) but significantly lower in the RA group than in the control group (*P* < 0.001). RA administration reduced the ICP0-induced increase in IL-17A protein levels (*P* = 0.0208).

The IL-23 protein levels showed no statistically significant difference between the control and ICP0 groups (*P* = 0.3272) and the RA+ICP0 group (*P* = 0.5353). IL-23 protein levels were significantly higher in the RA group than in the control (*P* = 0.0053), ICP0 (*P* = 0.0007), and RA+ICP0 groups (*P* = 0.0336).

TLR3 mRNA expression levels

To determine the TLR3 mRNA expression levels, ICP0 and RA were applied to the ARPE-19 cell line at predetermined IC50 dose concentrations. The mRNA expression levels were evaluated after 24 hours (Table 3).

**Table 3:** Alterations of the relative mRNA expression levels of TLR3 gene in ARPE-19 cell line after treatment with ICP0 and ICP0+RA indicated. The cells were treated with 277 µM ICP0 alone (24 hours), and 424.3 µM RA plus 277 µM ICP0 combination (24 hours), respectively. mRNA expression levels of these genes were normalized to GAPDH. ^*^Differences were considered to be significant compared to the control group (*P *< 0.05). ICP0, infected cell protein 0; RA, retinoic acid; GAPDH, glyceraldehyde 3-phosphate dehydrogenase; TLR3, toll-like receptor 3

Genes	ICP0			ICP0+RA		
	Gene expression	*P*-value	Up-/downregulation	Gene expression	*P*-value	Up-/downregulation
TLR3	4.155	0.003^*^	Significant upregulation	0.717	0.419	Insignificant upregulation

**Figure 3:**
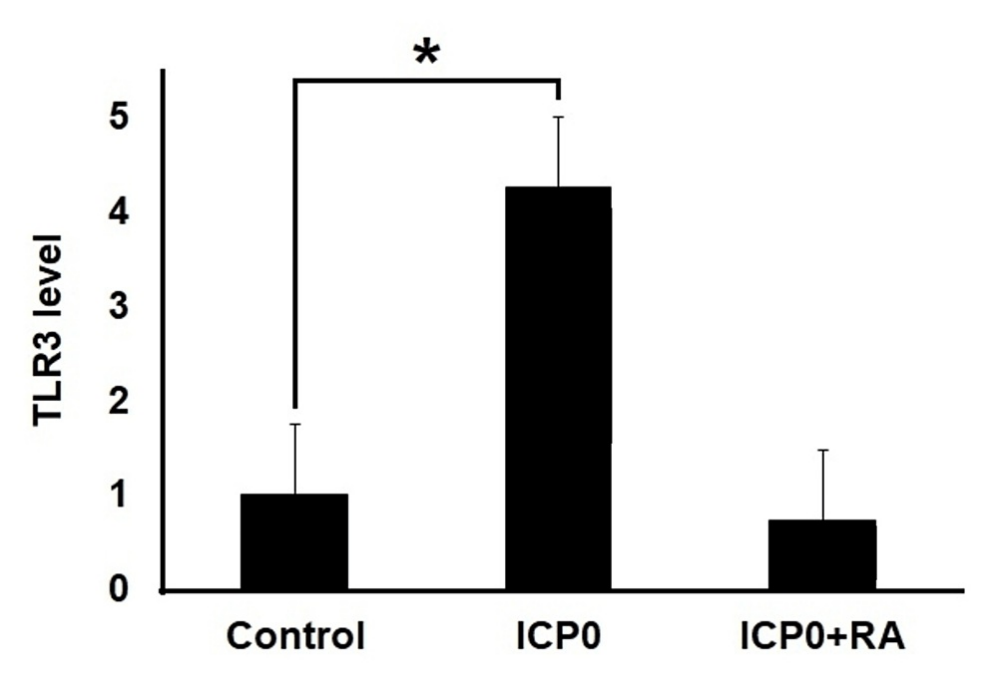
Alterations of the relative mRNA expression levels of TLR3 gene in ARPE-19 cell line after treatment with ICP0 and ICP0+RA indicated. The cells were treated with 277 µM ICP0 alone (24 hours) and 424.3 µM RA plus 277 µM ICP0 combination (24 hours), respectively. mRNA expression levels of these genes were normalized to GAPDH. ^*^Differences were considered to be significant in experiments when *P* < 0.05. ICP0, infected cell protein 0; RA, retinoic acid; GAPDH, glyceraldehyde 3-phosphate dehydrogenase; TLR3, toll-like receptor 3

TLR3 mRNA expression levels in the ICP0 and RA+ICP0 groups were 4.155 pg/mg and 0.717 pg/mg proteins, respectively (Figure 3).

## Discussion

HSK is the most common cause of corneal diseases leading to blindness in the United States and Europe, with a global incidence of approximately 1.5 million cases annually, resulting in approximately 40,000 new cases of severe monocular visual impairment or blindness [15]. In HSK, corneal edema and/or scarring occur depending on the pathogenic effect of the virus and the intensity of the host’s immune response, with stromal and endothelial HSK infections being the most severe and epithelial lesions being the most common [16].

HSV infection affects the eyelids, conjunctiva, cornea, uvea, and rarely the retina. In primary infection, ophthalmia neonatorum and blepharoconjunctivitis are observed. In recurrent infection, it causes blepharoconjunctivitis in adults, herpetic keratitis, marginal keratitis, necrotizing stromal keratitis, metaherpetic keratitis, endotheliitis, keratouveitis, and acute retinal necrosis. In our study, ICP0 was used to create an infection-like effect for HSK. Because ICP0 functions as a multifunctional transcriptional activator of cellular and viral genes in HSV-1 infection [17].

ARPE-19 cells had a very high IC50 value for RA in our study. On the other hand, Wang et al. observed the RA IC50 value as 140 μM in RAW264.7 cells [18]. Hamad et al. determined the IC50 value as 140 μM in Caco-2 and HCT-116 cells [19]. Dastjerdi et al. found the IC50 value to be 50 μM in OVCAR-3 cells [20].

IL-17A exhibits proinflammatory effects by activating neutrophils and other immune cells, inducing tumor necrosis factor (TNF)-α, IL-1β, IL-6, CXC chemokines, and chemoattractants [21]. Our study showed that the IL-17A level in the control group was 289.4 pg/mg protein after 24-hour exposure of ARPE-19 cells to HSV-1 ICP0, but it increased significantly to 410.7 pg/mg protein in the ICP0 group (*P* = 0.001), indicating that ICP0 may have antigenic effects, thereby activating the immune system.

Kim et al. suggested that IL-17A suppression may result in robust Th1 cell development following infection, potentially eliminating and preventing the virus from reaching the ganglia [22]. In the present study, IL-17A levels were lower in the RA group than in the control group (*P* < 0.001). RA administration decreased the ICP0-induced increase in IL-17A levels to 166.9 pg/mg protein (*P* = 0.0208) (Figure 2 and Table 2).

IL-1α, IL-6, IL-17A, and matrix metalloproteinase-9 contribute to the development of corneal neovascularization in response to HSV-1 infection [23]. Our findings showed that RA decreased the ICP0-induced increase in IL-17A levels, indicating that RA may prevent corneal hemangiogenesis caused by HSV-1.

Notably, RA suppressed IL-17A, which plays an important role in tissue damage. IL-17A is rapidly produced during the early stages of HSV-1 infection, and its primary sources include innate cells, particularly γ/δ T cells [24]. Neutrophil migration is associated with corneal opacity in HSV infection, indicating that IL-17 plays a role in tissue damage [25].

IL-23 plays a role in Th17 cell survival and proliferation [26, 27]. Our study showed that IL-23 levels were similar between the ICP0 and control groups (p = 0.3272), but a significant difference was found between the ICP0 and RA+ICP0 groups (*P* = 0.0484) (Figure 2 and Table 2), indicating that RA can significantly increase IL-23 levels even in the presence of ICP0.

In dry eye disease, IL-23, IL-6, and TGF-β are significantly upregulated in the cornea and conjunctiva [28]. As a secondary outcome of the study, IL-23 levels significantly increased in the RA group compared with the other groups, indicating the potential use of RA in the treatment of dry eye disease (Figure 2 and Table 2).

Within the first 24 hours following HSV-1 infection, chemokines mediated by TLR3 are produced in the cornea, initiating an immune response that directs the migration of natural killer cells and inflammatory monocytes to the cornea [29]. Compared with the control group, a 4155-fold increase in TLR3 mRNA expression was detected in ARPE-19 cells 24 hours after exposure to ICP0, which was consistent with the literature (*P* = 0.003) (Table 3).

Duncan et al. reported that pre-exposure of ARPE cells to viruses could render them susceptible to subsequent TLR ligand exposure or viral exposure, potentially leading to excessive cytokine release (cytokine storm) [30]. In the present study, while ICP0 dramatically increased TLR-3 mRNA expression compared with the control group, the combination of RA and ICP0 returned TLR-3 mRNA expression to a level similar to that in the control group (*P* = 0.419) (Table 3). Therefore, RA can potentially prevent excessive cytokine release induced by HSV-1 infection, but additional studies are required to investigate this further.

The main limitation of our study is that IL-17A and IL-23 amounts were evaluated only at the 24th hour. To observe the change rates in more detail, examinations could be made at 12, 18, and 36 hours. Another limitation of the study is that the ICP0 protein was not isolated in our laboratory but was obtained commercially.

## Conclusions

Our study demonstrates the importance of RA for combined therapy in HSV-1 ocular infections. It was observed that RA neutralized the negative effects of the ICP0 virus protein. Thus, RA has a potential usage as a support to antimicrobials. Although RA can potentially neutralize the detrimental effects of the HSV-1 ICP0 in ARPE-19 cells, comprehensive studies are necessary to elucidate the immunomodulatory effects of RA and its effects on antiviral resistance.
